# Effect of Pre-Procedural Antiseptic Mouthwash On The Dentin Bond Strength of Dental Adhesives

**DOI:** 10.3290/j.jad.c_1854

**Published:** 2025-02-04

**Authors:** Sutasinee Srichai, Pipop Saikaew, Vanthana Sattabanasuk, Pisol Senawongse

**Affiliations:** a Sutasinee Srichai Faculty of Dentistry, Mahidol University, Department of Operative Dentistry and Endodontics, Bangkok 10400, Thailand. Conceived and executed the study; wrote the manuscript.; b Pipop Saikaew Faculty of Dentistry, Mahidol University, Department of Operative Dentistry and Endodontics, Bangkok 10400, Thailand. Idea, substantial to discussion, proofread the manuscript.; c Vanthana Sattabanasuk Faculty of Dentistry, Mahidol University, Department of Operative Dentistry and Endodontics, Bangkok 10400, Thailand. Idea, substantial to discussion, proofread the manuscript.; d Pisol Senawongse Faculty of Dentistry, Mahidol University, Department of Operative Dentistry and Endodontics, Bangkok 10400, Thailand. Proofread the manuscript.

**Keywords:** adhesion to dentin, adhesive, dentin, microtensile bond strength, pre-procedural antiseptic mouthwash

## Abstract

Objective: To evaluate the effect of pre-procedural antiseptic mouthwashes on dentin bond strength of different adhesive systems.

Methods: Flat occlusal dentin surfaces from 120 extracted human molars were randomly divided into four groups according to mouthwashes (0.12% chlorhexidine = CHX, 1% hydrogen peroxide = HP, 0.2% povidone-iodine = PI, and no mouthwash/control) and three subgroups of adhesives used (Clearfil SE Bond; CSE, Single Bond Universal = SBU in etch-and-rinse (ER) or self-etch (SE) modes) (n = 8). Composite resin was built up, and all bonded teeth were stored in 37°C distilled water for 24 h. Stick-shaped specimens were prepared and subjected to microtensile bond strength (µTBS) test. Failure mode analysis was determined using a light microscope. A resin-dentin interface was observed using scanning electron microscopy (SEM, n = 2). Elemental analysis in the PI group was further examined by SEM with energy-dispersive X-ray spectroscopy. The µTBS data were statistically analyzed by two-way analysis of variance (ANOVA) and Duncan’s multiple comparison (P < 0.05).

Results: Rinsing with PI followed by SBU-SE demonstrated significantly higher µTBS than the control group (P < 0.05). Rinsing with HP showed significantly lower bond strength for CSE (P < 0.05). However, the effect of adhesive systems was not observed for all mouthwashes used (P > 0.05). SEM/EDX revealed the iodine deposition in the underlying dentin, where the highest amount of iodine was found for SBU-SE.

Conclusion: CHX and PI can be recommended as pre-procedural antiseptic mouthwashes since they show no negative impact on µTBS for all tested adhesives. The dentin bond strength of CSE is hampered in the HP mouthwash group, and this should be a concern for the use of self-etching adhesive afterward.

At the end of 2019, a novel coronavirus designated as severe acute respiratory syndrome coronavirus 2 (SARS-CoV-2), emerged in the city of Wuhan, China, and caused an outbreak of an unusual viral pneumonia.^15^ The disease was named coronavirus disease 2019 (COVID-19) by the World Health Organization (WHO)^14^ and spread rapidly all over the world. A high SARS-CoV-2 viral load has been detected in COVID-19-positive patients’ saliva^23^ and periodontal pockets.^4^ Many attempts were made to prevent the viral transmission, eg, postponing treatment for patients suspected of being infected with COVID-19, using infection control measures such as standard personal protective equipment (PPE) and extraoral vacuum aspirator.^34^

In 2020, the American Dental Association (ADA) and the Center for Disease Control and Prevention (CDC) recommended the use of pre-procedural antiseptic mouthwashes, eg, chlorhexidine (CHX), hydrogen peroxide (HP), and povidone-iodine (PI) before dental procedures.^40^ In a clinical study, the standard infection control using pre-procedural antiseptic mouthwashes and intraoral high-volume evacuation were considered sufficient and reduced the risk of viral transmission to the operators and patients.^27^

Bonding to dentin is challenging due to the complexity of dental substrate. Clearfil SE Bond (Kuraray Noritake, Tokyo, Japan) is considered the “gold standard” self-etching adhesive with 10-methacryloyloxydecyl dihydrogen phosphate (10-MDP) as a functional monomer.^26^ The phosphate group in 10-MDP promotes the chemical interactions that form a water-insoluble salt with hydroxyapatite. This 10-MDP-Ca salt contributes to collagen fiber protection and bond stability improvement.^5^ In late 2011, the recent classification of adhesive, referring to the 8th generation or universal adhesive, was introduced. This adhesive mainly contains 10- MDP, which can be used in etch-and-rinse, self-etch, and selective enamel etching modes.^12^

The effects of the pre-procedural antiseptic mouthwashes on dentin bond strength are not consistent across publications. Many studies found that using 2% CHX before acid-etching did not negatively affect the dentin bond strength of etch-and-rinse adhesives,^19,31^ whereas this procedure improved the bond strength of self-etch adhesives.^16^ However, the bond strength of adhesives reduced when 3% HP was used before adhesive application, regardless of the adhesive system employed.^9,38^ In contrast, the bond strength of etch-and-rinse adhesive was higher after rinsing with 10% PI.^1^ Regarding the bonding performance of universal adhesive to dentin, there is still insufficient information on the effect of different pre-procedural antiseptic mouthwashes. A previous study has evaluated the effect of antiseptic mouthwashes on the universal adhesive, but only HP and PI were used.^21^ The study by Shirani et al assessed several mouthwashes, however only 1 two-step self-etch adhesive was tested.^36^ Therefore, the purpose of this study was to evaluate the effect of three different pre-procedural antiseptic mouthwashes on the dentin bond strength of a universal adhesive in different application modes compared with that of Clearfil SE Bond. The null hypotheses were (1) there was no effect of the pre-procedural antiseptic mouthwashes on the dentin bond strength; (2) there was no difference in dentin bond strength between adhesive systems.

## MATERIALS AND METHODS

### Tooth Selection and Specimen Preparation

One hundred and twenty extracted human third molars without carious lesions, cracks, or restorations were collected following a protocol that was approved by the University Ethics Committee. The collected teeth were sectioned perpendicular to the long axis of the tooth using a low-speed diamond saw (Diamond blade 4-inch series HC, PACE Technologies, Tucson, AZ, USA) until the mid-coronal dentin was exposed. The dentin surface was then polished using #600 grit silicon carbide (SiC) paper for 60 s.^20^ The teeth were randomly divided into 12 groups (n = 10) based on the pre-procedural antiseptic mouthwashes: no mouthwash (control); chlorhexidine (CHX); hydrogen peroxide (HP); and povidone-iodine (PI), and adhesive systems: Single Bond Universal applied in etch-and-rinse mode (SBU-ER), self-etch mode (SBU-SE), and Clearfil SE Bond (CSE). The protocols for each mouthwash are provided in Table 1. The concentrations of the mouthwashes were used per the CDC recommendations. The compositions of the adhesives and their application steps are demonstrated in Table 2. The adhesives were always tightly closed and kept refrigerated. Just only before use, they were removed from the refrigerator and left at room temperature for 30 min. Each adhesive was dropped in the sterile mixing plate and manipulated immediately with a disposable applicator. After adhesive application, the adhesive layer was light-cured with a light curing unit (Blue phase G2, Ivoclar Vivadent, Schann, Liechtenstein) for 10 s. The light intensity, which was not less than 1,000 mW/cm^2^, was periodically checked. Each bonded tooth was built up with a composite resin (Clearfil AP-X ES-2, Kuraray Noritake Dental, Tokyo, Japan). A flat, surface-coated composite instrument was used to press a first 2 mm-thick layer of composite resin to ensure the adaptability of the material especially at the central area. Another layer was then built up to reach a 4-mm thickness with separated photopolymerization each increment for 20 s. The resin-bonded teeth were stored in distilled water at 37°C for 24 h.

**Table 1:** Protocols of each pre-procedural antiseptic mouthwash

Control	Tooth surfaces were rinsed with distilled water for 30 s.
CHX (pH 6.07)	Immersed in 10 ml of 0.12% CHX solution for 30 s, then rinsed with distilled water for 30 s.
HP (pH 4.55)	Immersed in 10 ml of freshly mixed 1% H_2_O_2_ solution for 30 s, then rinsed with distilled water for 30 s.
PI (pH 3.02)	Immersed in 10 ml of 0.2% PI solution for 30 s, then rinsed with distilled water for 30 s.

**Table 2:** Compositions and applications of the adhesives used in the study

Single Bond Universal (3M Oral Care, St. Paul, MN, USA)	10-MDP, HEMA, dimethacrylate resins, vitrebond copolymer, filler, ethanol, water, initiator, silane	Etch-and-rinse mode 1. Apply etchant for 15 s 2. Rinse for 15 s 3. Air dry to remove excess water by keeping dentin moist 4. Apply adhesive and rub it for 20 s 5. Gently air dry for 5 s 6. Light-cure for 10 s
Self-etch mode 1. Apply adhesive and rub it for 20 s 2. Gently air dry for 5 s 3. Light-cure for 10 s
Clearfil SE Bond (Kuraray Noritake Dental, Tokyo, Japan)	Primer: 10-MDP, HEMA, hydrophilic aliphatic dimethacrylate, dl-camphorquinone, water Bond: 10-MDP, Bis-GMA, HEMA, hydrophobic aliphatic dimethacrylate, dl-camphorquinone, initiators, accelerators, silanated colloidal silica	1. Apply primer and leave for 20 s 2. Dry thoroughly with mild air blow 3. Apply bonding 4. Gently air blow 5. Light-cure for 10 s
Abbreviations: 10-MDP = 10-methacryloyloxydecyl dihydrogen phosphate; HEMA = hydroxyethyl methacrylate; Bis-GMA = bisphenol-A glycidyl dimethacrylate

### Microtensile (µTBS) Bond Strength Test

Eight resin-bonded teeth per group were used for the µTBS test.^3^ Each tooth was cut to achieve resin-dentin stick-shaped specimens with cross-sectional area ~1 × 1 mm^2^. Nine sticks from the central area of the tooth were selected. The µTBS test was performed using a universal testing machine (Lloyd Testing Machine, Model LR 10K, Lloyd Instruments, Fareham Hanth, UK) with a cross-head speed of 1 mm/min. The µTBS values from each bonded tooth were averaged and used for statistical analysis.^3^ The fractured specimens were left to dry overnight in an incubator at 37°C. The specimens were mounted onto metal stubs for failure mode analysis using a light microscope at 40×(DP22, Olympus, Tokyo, Japan). The failure modes were classified into four types: (1) cohesive in dentin; (2) adhesive failure; (3) cohesive in composite resin; and (4) mixed failure.^35^

### Resin-dentin Interface Observation

Two resin-bonded teeth from each group were used for observation of the resin-dentin interface (n = 2). An ~1-mm-thick resin-dentin slab was sectioned in the buccolingual dimension. The slabs were embedded in the epoxy resin and polished with #600 to #1,200-grit SiC paper under running water, and 6, 3, 1, and 0.25 µm particle size diamond pastes.^10^ The specimens were etched with 10% phosphoric acid for 5 s, washed, and treated with 5.25% NaOCl for 10 min.^28^ After storage in an incubator at 37°C for 24 h, the embedded specimens were coated with palladium (K500X Sputter coater, SPI supplies, West Chester, PA, USA) and observed using a scanning electron microscope (SEM, JSM 6610LV, JEOL, Peabody, MA, USA) at 3,000× magnification.

### Elemental Analysis of the Resin-dentin Interface With SEM/EDX

After observation by SEM, all specimens in the PI group were further examined for their calcium, phosphorus, silicon, and iodine contents. SBU-SE specimen in the control group with no mouthwash rinsing was also investigated. The deposition of these ions on the resin-dentin interface was determined by elemental mapping analysis and elemental line scan at the selected line via energy-dispersive X-ray spectrometry (EDX) using SEM. The line was randomly selected for 60 µm in length from composite resin toward the dentin.

### Statistical Analysis

The statistical analysis was performed using SPSS Statistics 23 (SPSS, Chicago, IL, USA). The data were organized, and the homogeneity of variance and normal distribution were analyzed using the Shapiro-Wilk test and Levene test, respectively. The effects of the pre-procedural antiseptic mouthwashes and adhesive systems were calculated using two-way analysis of variance (ANOVA) and followed by Duncan’s multiple comparison. The P-value threshold for significance was set at 0.05.

## RESULTS

### Microtensile Bond Strength

Two-way ANOVA revealed the significant effect of the different mouthwashes (P < 0.001, observed power = 0.988) and the interaction between adhesive and mouthwash (P = 0.022) on the bond strength values; however, the effect of the adhesive groups was not significant (P > 0.05).

The means µTBS and standard deviations (MPa) are shown in Table 3. No pre-test failure occurred in this study. The highest bond strength was observed in the PI group with SBU-SE. In contrast, the lowest bond strength was found in the HP group with CSE. A significant effect of the mouthwash was detected in the SBU-SE and CSE groups. Compared with the control, rinsing with PI resulted in increased µTBS of SBU-SE, while rinsing with HP led to decreased CSE µTBS.

**Table 3:** Mean µTBS with standard deviation (MPa) and failure mode distribution (number of stick-shaped specimens)

**Single Bond Universal (ER)**	26.78 + 7.07^a^ 20/6/7/39	27.23 + 3.35^a^ 40/2/1/29	29.51 + 6.40^a^ 24/3/4/41	31.07 + 4.74^a^ 18/3/5/46
**Single Bond Universal (SE)**	25.41 + 4.46^a, b^ 31/2/2/37	29.10 + 4.71^b, c^ 23/2/3/44	23.02 + 5.15^a^ 29/3/1/39	33.46 + 4.66^c^ 25/2/1/44
**Clearfil SE Bond**	30.65 + 6.93^a, b^ 17/1/3/51	26.34 + 2.27^a, c^ 31/2/4/35	22.65 + 5.11^c^ 16/7/1/48	32.89 + 6.09^b^ 20/2/4/46

### Failure Mode Analysis

The result of the failure mode analysis is also presented in Table 3. The failure mode was mainly categorized as adhesive failure, followed by cohesive failure in the composite. The failure mode distributions were similar in all experimental groups.

### Resin-dentin Interface Observation

**Fig 1:**
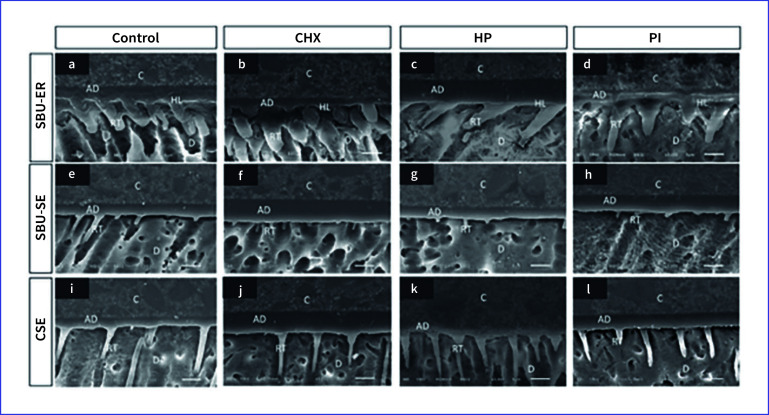
The representative resin-dentin interface of each adhesive system with different pre-procedural antiseptic mouthwashes.Abbreviations: CHX = chlorhexidine; HP = hydrogen peroxide; PI = povidone-iodine; C= composite resin; D = dentin; AD = adhesive; HL = hybrid layer; RT= resin tags; SBU-ER = Single Bond Universal (etch-and-rinse); SBU-SE = Single Bond Universal (self-etch); CSE = Clearfil SE Bond.

Representative images of the resin-dentin interface are demonstrated in Fig 1. The images of the different mouthwashes for each adhesive group were similar (Fig 1a–d, 1e–h, and 1i–l). SBU-ER demonstrated large, conical resin tags that were more abundant (Fig 1a–d) compared with the other groups. The hybrid layer, approximately 1–2 µm in thickness, can be detected only in the SBU-ER group. The SBU-SE and CSE groups showed similar characteristics. Resin tags of the SBU-SE groups were cylindrical and fewer in number (Fig 1e–h); however, those of the CSE groups were longer (Fig 1i–l).

### Elemental Analysis of Resin-dentin Interface With SEM/EDX

Table 4 presents the elemental analysis of iodine in weight% and atomic% for the specimens in the PI group with different adhesives tested. Iodine could not be detected in the control group. With the use of PI mouthwash, the highest amount of iodine was found in the SBU-SE group at 0.97 weight% and 0.13 atomic%. The SBU-ER group demonstrated the lowest amounts of iodine deposition.

**Table 4:** Elemental analysis of iodine with different adhesive systems

**Control (no mouthwash)**	Not found	Not found
**PI and bonded with Single Bond Universal (ER)**	0.31	0.04
**PI and bonded with Single Bond Universal (SE)**	0.97	0.13
**PI and bonded with Clearfil SE Bond**	0.71	0.10
		

**Fig 2:**
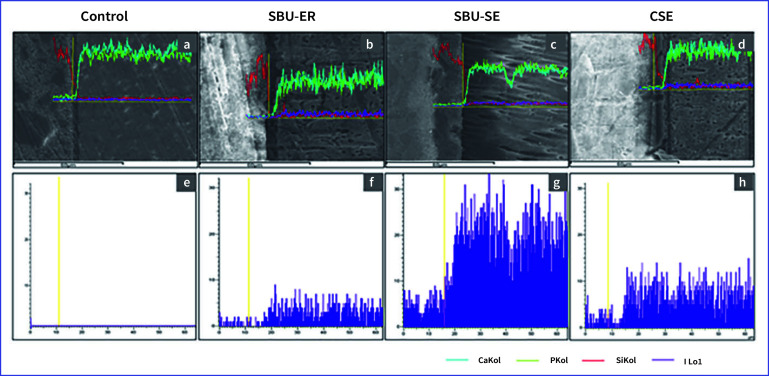
The elemental line scans of the resin-dentin interface of each group that were rinsed with povidone-iodine.Abbreviations: SBU-ER = Single Bond Universal (etch-and-rinse); SBU-SE= Single Bond Universal (self-etch); CSE = Clearfil SE Bond; Ca = calcium; P = phosphorus; Si = silicon; I = iodine.

The elemental line scans of the resin-dentin interface are shown in Figure 2. Calcium and phosphorus ions were distinct in dentin, whereas silicon was largely presented in the composite resin and the adhesive layer (Fig 2a–2d). Except for the control with no mouthwash used, iodine deposition was notably detected for all adhesive groups, especially in the adhesive layer and dentin (Fig 2e–2h).

The iodine deposition was highest in the SBU-SE (Fig 2c and 2g), followed by CSE (Fig 2d and 2h) at ~30 and 15 X-ray counts, respectively. In contrast, a lower X-ray count of iodine in dentin was observed in the SBU-ER group (Fig 2b and 2f).

## DISCUSSION

The results of this study indicated that the pre-procedural antiseptic mouthwashes differently affected the dentin bond strength of the evaluated adhesives (Table 3). CHX did not influence the µTBS in all adhesive groups. PI significantly increased the bond strength of SBU-SE (P < 0.05). However, HP had a negative effect on the dentin bond strength of SBU-SE and CSE (P < 0.05). Thus, the first null hypothesis was not accepted. On the contrary, the effect of the adhesive system on the bond strength was not significant (P > 0.05). Therefore, the second null hypothesis was accepted.

The three different mouthwashes used in this study (CHX, HP, PI) were selected based on the CDC’s recommendation. PI appears to have a positive effect on bond strength, showing a clear trend toward enhancement. Conversely, HP seems to negatively influence the bond strength, displaying a tendency to weaken the adhesive bond. CHX, on the other hand, maintains a neutral impact, with stability of dentin bond strength observed.

A significant improvement in bond strength was observed when rinsed with PI in the SBU-SE group. PI is a water-soluble polymer composed of an iodine and the polyvinyl pyrrolidone, which has a complex affinity for both hydrophilic and hydrophobic polymers^1^, however, its structure containing a polar amide and methylene, makes it highly soluble in aqueous and non-aqueous solutions.^41^ Its mechanism is releasing free iodine, which disrupts microbial metabolic pathways.^11^ It is not clear how PI improved the bonding performance of SBU-SE. A plausible explanation could be that the slight acidity of PI with pH at 3.02 (Table 1) might enhance the etching effect of SBU-SE, which is an ultra-mild adhesive.^39^ We also speculated that the residual free iodine in the underlying dentin might play an important role in the bonding performance of the adhesive. Our elemental analysis demonstrated that the amount of iodine detected was highest in the SBU-SE group (Table 4, Fig 2g) followed by the CSE (Fig 2h) and SBU-ER groups (Fig 2f). The phosphoric acid-etching and rinsing in the SBU-ER group and the primer application in the CSE group might have eliminated the remaining free iodine. The results are in line with Alamoudi et al who used a 10% PI pretreatment on the dentin and found that there was no significant effect of PI on the microtensile bond strength of etch-and-rinse adhesive.^1^ These could be the reasons why the bond strengths of SBU-ER and CSE were not affected by PI in the current observation. Further research is required to shed more light on this topic.

CHX did not influence the bond strength of the adhesives tested. This agrees with the previous studies.^17,19,31,37,42^ No effect of CHX was observed for the immediate bond strength. However, it was reported that CHX has a high affinity to dentin by binding to the phosphate groups of dentin and carboxyl groups of collagen fibrils.^18^ Furthermore, the application of CHX could have delayed the bonding degradation by inhibiting matrix metalloproteinase activity.^19^ Thus, the benefit of rinsing with CHX might be more apparent over the long-term dentin adhesion.

The adverse effect of pre-procedural antiseptic mouthwash was observed in the HP group. Compared with the control, lower bond strengths were found for the CSE and SBU-SE groups, with a significant effect detected for the CSE group (Table 3). The negative effect may be due to the hydroxyl free radicals (-OH) produced by HP remaining in the collagen matrix, which attack several cell components, such as proteins and DNA,^13^ and interfere with resin infiltration and polymerization.^32^ Furthermore, HP molecules have been associated with morphological alterations and changes in dentin compositions.^22^ Therefore, the mechanical properties of the dentin substrate could be aggravated.^6^ The results of the present study were similar to another investigation, which demonstrated that the treatment of 3% HP on a dentin surface for 20 s reduced the bond strength of self-etching adhesive. However, no adverse effect was found when the etch-and-rinse adhesive was used.^9^ Similarly, Shirani et al reported that treatment of dentin with 1% HP for 60 s, followed by rinsing under strong water flow, did not significantly change the bond strength of self-etching adhesive used.^36^ In addition to the use of low-concentration HP mouthwash, copious rinse with water or the prior application of phosphoric acid might help eliminate the remaining free radicals on the dentin surface and reduce the adverse effect of HP.

The bond strength was not significantly different between the adhesives used. Rosa et al^33^ reported that there was no significant difference in the dentin microtensile bond between the etch-and-rinse and self-etch strategies for mild universal adhesives. The adhesives used in the present study were Single Bond Universal or Scotchbond Universal (pH = 2.7) and Clearfil SE Bond (pH = 2) that are classified as an ultra-mild and mild adhesive, respectively.^39^ Likewise, Costa et al reported that the tensile bond strength between Scotchbond Universal and Clearfil SE Bond was not significantly different.^7^ Upon closer inspection, SBU-ER provided higher dentin bond strength than SBU-SE and CSE for the HP group, even though the statistical difference could not be detected. As stated previously, the application of phosphoric acid in etch-and-rinse mode of SBU could have reversed the compromised bond strength of HP-treated dentin.^9^ HP, as a pre-procedural antiseptic mouthwash, should then be used cautiously if the self-etch adhesives are selected in clinical works.

In this study, the pre-procedural antiseptic mouthwashes were applied, irrigated with distilled water, and then restored with composite resin. However, this might not simulate the clinical situations where the cavity preparation is required. Thus, the effect of the mouthwashes might be minimized. However, this study’s protocol simulated the situation when clinicians restore non-carious cervical lesions (NCCLs). The optimum surface preparation technique for NCCLs has not been determined. Luhrs et al^24^ reported that restorations placed on the dentin surface without any preparations had the lowest retention rate. In the present study, we investigated the direct effects of antiseptic mouthwashes on dentin adhesion. Thus, the dentin surfaces were prepared without cleaning with pumice or grinding with a rotary bur.

## CONCLUSIONS

Within the limitations of this study, these following conclusions can be drawn.

Rinsing with pre-procedural antiseptic mouthwash affects dentin bond strength in different ways, in which PI increases the bond strength of SBU in SE mode, HP decreases bond strength of CSE, while CHX has no effect on the bond strength of all adhesives tested.
Maximum bond strength of SBU is acquired when used in SE mode with PI as a pre-procedural antiseptic mouthwash. HP is not recommended when bonded with CSE.
